# Spontaneous Isolated Symptomatic Celiac Artery Dissection: A Case Report

**DOI:** 10.7759/cureus.85960

**Published:** 2025-06-13

**Authors:** George S Zacharia, Anandu M Anto, Faryal Altaf, Anshuman Sikka, Misbahuddin Khaja

**Affiliations:** 1 Internal Medicine, BronxCare Health System, Bronx, USA; 2 General Surgery, BronxCare Health System, Bronx, USA

**Keywords:** acute abdomen, arterial dissection, celiac artery aneurysm, hepatitis, pancreatitis

## Abstract

The celiac artery, also known as the celiac trunk, originates from the abdominal aorta at the level of the 12th thoracic vertebra. Arising from the anterior aorta, this ultrashort artery plays an integral role in supplying blood to the stomach, liver, spleen, pancreas, and proximal duodenum. Arterial dissection is a life-threatening emergency most frequently reported in the aorta, while visceral arterial dissections remain uncommon or may be undetected. A dissection involves a tear in the artery wall, which can lead to serious complications, including compromised arterial supply, end-organ ischemia, and rupture. Spontaneous dissection of the celiac artery is a rare vascular disease. Most patients are asymptomatic and incidentally diagnosed on abdominal imaging, but can present as acute abdominal pain. A dissection of the celiac trunk could potentially lead to a disruption in the perfusion of the pancreas, liver, and stomach with subsequent development of ischemia, inflammation, and infarction. This is, however, an uncommon event and has been described only in very few cases in the past. Our case describes this uncommon presentation, a patient with features of pancreatitis and hepatitis in the setting of a celiac artery dissection, likely related to ischemia. The diagnosis of celiac artery dissection relies on computerized tomography (CT) with contrast, preferably with angiography. Medical management, endovascular therapy with stents and rarely surgical reconstruction, or bypass could be employed for treating symptomatic patients. Ironically, endovascular treatment for cardiac and vascular diseases has emerged as a frequent culprit in the causation of arterial dissection: iatrogenic arterial dissection.

## Introduction

Spontaneous isolated celiac artery dissection (SICAD) is an exceedingly rare scenario, with only less than 500 cases reported in published medical literature to date [1]. The celiac artery is the first branch of the abdominal aorta and is pivotal to arterial supply to the stomach, duodenum, liver, pancreas, and spleen. Hence, a compromise of the artery, especially an acute event, can inflict variable degrees of ischemic injury to these vital organs. Depending on the severity and acuity of dissection and the extent of collateral circulation, SICAD can present with or without symptoms. Asymptomatic dissections are incidentally detected during abdominal imaging. Symptomatic patients often experience acute or chronic abdominal pain, which can be severe and persistent due to organ ischemia. The symptomatology may also include nausea, vomiting, and other features of organ dysfunction. Symptomatic cases are more likely to be diagnosed promptly because of the acute symptoms. Modern high-resolution imaging techniques, CT, or magnetic resonance imaging (MRI), especially when contrast-enhanced, allow identification of dissection with the demonstration of an intimal flap or an eccentric mural thrombus and thereby accurate diagnosis. The treatment strategies essentially aim at preventing complications such as ischemia, aneurysm formation, and rupture. There are no standard and universally accepted recommendations on managing SICAD, and the treatment is somewhat tailored to dissection symptoms, severity, and complications. Conservative management involves monitoring and medical therapy, primarily for asymptomatic patients or those with mild symptoms [2]. Endovascular intervention is considered for patients with significant symptoms or complications. This minimally invasive procedure often involves placing a stent to stabilize the artery and restore blood flow, offering a less invasive alternative to surgery [2]. Surgical interventions are reserved for severe cases where endovascular procedures are not feasible, have failed, or have a critical complication like rupture and involve direct surgical correction or bypass of the dissection [2].

## Case presentation

A 54-year-old male with a history of hypertension and hyperlipidemia presented with abdominal pain and dizziness. He was asymptomatic till the day of the current presentation. He reported severe, constant epigastric pain associated with two episodes of non-bilious, non-hemorrhagic vomiting with subsequent dizziness and generalized weakness. He had no overt gastrointestinal bleeding, diarrhea, fever, or icterus. He denied any history of trauma, falls, or surgery in the recent past but had a cholecystectomy for gallstone disease six years back. He declined the use of ethanol or other substances of abuse. On physical exam, he was hemodynamically stable but appeared in modest distress. He was warm, with no evidence of scleral icterus, and mucus membranes were moist without erythema or exudate. The abdomen was soft, non-distended, and mildly tender to palpation in the epigastrium, with no rigidity or guarding. The rest of the clinical examination was within normal limits. The laboratory workup revealed transaminases and lipase exceeding 10 times the standard upper limit, suggesting concomitant hepatic and pancreatic inflammation. Serum lactate dehydrogenase and lactic acid were elevated as well (Table 1).

**Table 1:** Admission laboratory data

Pertinent labs	Test values	Reference values
Hemoglobin	17.1	12-16 g/dl
Hematocrit	51.5	42-51
WBC	14.8	4.8-10.8 k/ul
Platelet	311	150-400 k/ul
Neutrophil %	87.2	40-70 %
Sodium, serum	137	135-145 mEq/L
Potassium, serum	5	3.5-5 mEq/L
Blood urea nitrogen, serum	16	8-26 mg/dL
Creatinine, serum	1.5	0.5-1.5 mg/dL
Albumin, serum	4	3.4-4.8 g/dl
Bilirubin, serum total	2	0.2-1.2 mg/dl
Bilirubin, serum direct	1.6	0.0-0.3 mg/dl
Alkaline phosphatase, serum	203	56-119 unit/L
Aspartate transaminase, serum	1111	9-48 unit/L
Alanine aminotransferase, serum	1517	5-40 unit/L
Lipase, serum	5512	<=61 U/L
Hepatitis B surface antigen	Non-reactive	Non-reactive
Hepatitis B surface antibody	Non-reactive	Non-reactive
Hepatitis B core total antibody	Non-reactive	Non-reactive
Hepatitis C antibody (HCV)	Non-reactive	Non-reactive
Hepatitis D antibody, total	Negative	Negative
Lactic acid	2.5	0.5-1.6 mmoles/L
LDH	>1200	140 to 280U/L

**Figure 1:**
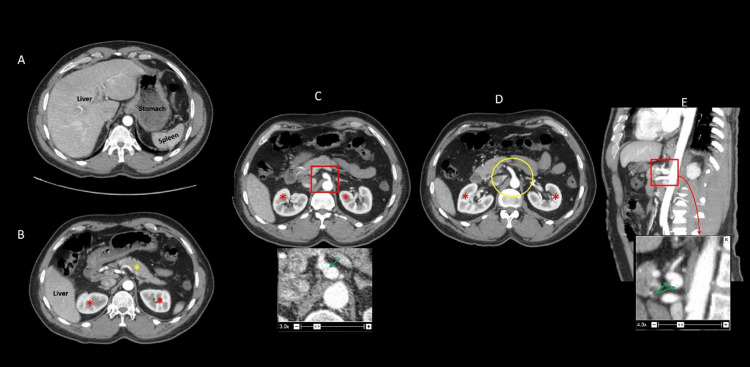
Computerized tomography (CT image). (A, B): Axial images with apparently normal-appearing liver, stomach, spleen, pancreas (yellow asterisk), and kidneys (red asterisk). (C) Axial image depicting the bulbous dilatation of the celiac artery trunk, which upon magnification demonstrates the intimal flap (green arrow). (D) Axial image showing the normal origin of the superior mesenteric artery. (E) Sagittal image remonstrating the dilated celiac artery trunk with an intimal flap (green arrow), while the normal superior mesenteric artery is visualized below.

A contrast-enhanced CT of the abdomen and pelvis showed that the celiac axis was dilated at its origin, with a small dissection flap within it, approximately 6 mm in length (Figure 1).

The hepatic artery and splenic artery branches were patent and not affected. Imaging did not reveal any extension of the dissection to adjacent arteries or any other dissections in the imaged regions. There was no common bile duct or intrahepatic biliary radicle dilatation, nor any calculi in the bile ducts or pancreas. The rest of the visualized abdominal and pelvic viscera were imaged as usual. The patient was administered analgesics for pain relief and intravenous labetolol for hypertension; the vascular surgery and cardiology team were consulted for opinion. Given the lack of facilities for advanced vascular interventions, the patient was transferred to a higher center for further evaluation and management. Additional information about the patient's clinical course or follow-up is limited except for the verbal information from the vascular team that the patient was managed conservatively with analgesics, optimization of blood pressure, and aspirin, with which his symptoms and biochemical parameters improved.

## Discussion

Arterial dissection is characterized by a separation of the arterial wall layers, often incited by a tear in the intima, most frequently reported in the aorta, carotid, and vertebral arteries. Visceral artery dissection is an uncommon event, most often involving the superior mesenteric artery [3]. The dissections may be iatrogenic or spontaneous. Spontaneous dissection of the celiac artery is exceedingly rare, with only a handful of cases reported in the published medical literature to date. The first documented case of spontaneous celiac dissection dates back to 1959 by Foord and Lewis [4]. The postulated risk factors of spontaneous arterial dissection include male gender (male: female ratio of 5:1), advanced age (mean 55 years), hypertension, atherosclerosis, pregnancy, fibromuscular dysplasia, cystic medial necrosis, connective tissue diseases, and other rare inflammatory or infectious, or congenital disorders of the vascular wall [5]. Anatomically, the celiac artery is the first ventral branch of the abdominal aorta, a short 1 to 1.5 centimeters long artery, terminating by bifurcating to the common hepatic and splenic arteries. The common hepatic artery feeds the liver, biliary tree, pancreatic head, stomach, and proximal duodenum. The splenic branch supplies the spleen, stomach, pancreatic body, and tail [6]. Celiac artery compromise, hence, can compromise the arterial supply and potentially cause ischemic changes in the liver, pancreas, spleen, and upper gastrointestinal tract. The dissection can remain asymptomatic, but when symptomatic, severe upper abdominal pain resulting from ischemia of tissue/organs fed by the artery is the most common presentation. Chronic dissection can predispose to chronic mesenteric ischemia and intestinal angina, with post-prandial pain, often in the presence of simultaneous superior mesenteric arterial disease. In patients with isolated celiac artery dissection, small bowel ischemia is less frequent compared to isolated superior mesenteric arterial diseases [5].

The diagnosis of celiac artery dissection relies mainly on abdominal imaging; contrast-enhanced CT is the primary diagnostic modality. The pathognomonic imaging finding in CT is an intimal flap; however, it may not be appreciable in many cases [5]. An eccentric mural thrombus in the celiac artery lumen is yet another clue to the diagnosis of dissection [5,7]. Fatty infiltration around the celiac trunk is another predictor of celiac artery dissection [5]. Magnetic resonance and conventional angiography are alternative imaging modalities and demonstrate similar findings. Bauersfeld reported a cleavage plane between arterial intima and externa in patients with visceral artery dissection, unlike in the aorta, where the cleavage plane is more frequently between the layers of the intima itself [8]. Given the rarity of the disease, there is limited data or consensus regarding the management of celiac artery dissection. The goals of treatment include limiting the extension of dissection and preventing end-organ ischemia. Management of hypertension is the key to preventing the extension of the flap, thereby reducing the chances of tissue/organ hypoperfusion and rupture. Beta-blockers are considered the first-line medications for blood pressure control in patients with arterial dissection. Antiplatelets and anticoagulants prevent thrombus formation and/or thrombus extension and reduce the risk of ischemic complications. However, the optimal choice between antiplatelets versus anticoagulants as well as the duration of therapy are not definitively established, and decisions are often made on a case-by-case basis. Antiplatelets may be preferred in some cases, especially when there is a concern about bleeding risk or when the patient has other contraindications to anticoagulation. Medical management with antihypertensives and antiplatelets/anticoagulants, without interventions, is reported to be effective in managing spontaneous visceral artery dissections [9,10]. A literature search revealed anticoagulant treatment: warfarin bridged with heparin during the initial phase in patients with celiac dissection to prevent thromboembolic complications [5,11]. Those patients failing conservative medical management should be considered for interventions, such as surgery or endovascular therapy, the latter gaining more acceptance owing to comparable efficacy and being less invasive [5,7,12]. Surgical options include resection followed by reconstruction or bypass [5]. 

The natural history of spontaneous visceral artery, including celiac artery dissection, is largely unclear, especially due to the rarity of the disease. However, the emerging literature suggests that the prognosis is not as dismal as believed, and it is not uncommon to encounter a self-limiting course. Medical management has yielded promising results in most cases. However, the progression of dissection with or without luminal thrombus formation could result in organ ischemia or even spontaneous rupture with catastrophic outcomes. There is a lack of clear consensus on when to treat, what to use for treatment, or how long to treat. Extrapolating the benefits of blood pressure control in aortic or cervico-vertebral arterial dissection, this strategy has been adopted in visceral artery dissections as well, again with no specific data or consensus [9].

Our patient, a middle-aged male with cardiovascular comorbidities, hypertension, and hyperlipidemia, with no reported antecedent trauma or interventional procedure, presented with an acute abdomen. His labs were consistent with concomitant acute pancreatitis and hepatitis, while the contrast CT abdomen detected an isolated celiac artery dissection. The patient underwent cholecystectomy in the past, six years ago, and had no evidence of biliary dilatation or calculus disease to account for his hepatic or pancreatic disease. Neither he had hypertriglyceridemia nor hypercalcemia to account for the episode of pancreatitis. Considering the acute presentation, simultaneous hepatic-pancreatic involvement, and lack of alternate causes, it is highly likely that the acute ischemia from the celiac dissection precipitated the hepatitis and pancreatitis. Acute pancreatitis from celiac artery dissection has been reported in the literature. The presentation in these cases included abdominal pain and elevated amylase/lipase, similar to our case [10,13,14]. Moreover, ischemic hepatitis has been reported in the past with celiac arterial diseases, including celiac artery dissection and stenosis [15,16,17]. The high transaminase levels exceeding 1000 IU/L with elevated LDH and lactate also point to an ischemic etiology [18]. In the setting of acute hepatitis, a transaminase-to-LDH ratio of <1.5 is an additional marker of ischemic origin [18]. 

## Conclusions

Celiac artery dissection is a rare clinical scenario when symptomatic, presenting with an acute abdomen of epigastric localization. Being a key arterial provider to the hepatobiliary tree and pancreas, the celiac artery compromise can result in ischemic pancreatitis and hepatitis. This case highlights the importance of considering celiac artery dissection in the list of differentials in patients with acute abdomen and concomitant hepato-pancreatic dysfunction, especially with transaminases exceeding 1000 and disproportionately elevated lactate dehydrogenase. Using contrast-enhanced CT scans could increase the diagnostic yield in suspected celiac artery dissection cases by better delineating the intimal flap or an eccentric hematoma in the celiac trunk.
